# Online health information seeking behavior, healthcare access, and health status during exceptional times

**DOI:** 10.1016/j.jebo.2024.02.032

**Published:** 2024-04

**Authors:** Cinzia Di Novi, Matija Kovacic, Cristina Elisa Orso

**Affiliations:** a European Commission, Joint Research Centre (JRC), Via Enrico Fermi 2749, TP 267, 21027, Ispra, VA, Italy; b Department of Law, Economics, and Cultures, University of Insubria, Como, Italy

**Keywords:** Health information seeking behavior, Healthcare access, Health status

## Abstract

Online health information seeking behavior (e-HISB) is becoming increasingly common and the trend has accelerated as a result of the COVID-19 pandemic when individuals strongly relied upon the Internet to stay informed by becoming exposed to a wider array of health information. Despite e-HISB having become a global trend, very few empirical investigations have analyzed its potential influence on healthcare access and individuals’ health status. In this paper, we try to fill this gap. We use data from the second SHARE Corona Survey, supplemented with data from the previous 8th wave of SHARE, and estimate a recursive model of e-HISB, healthcare access, and individuals’ health status that accounts for individuals’ unobserved heterogeneity. Our findings suggest that e-HISB can empower individuals to better understand health concerns, facilitating improved health condition management. However, e-HISB can also trigger a chain reaction, as navigating vast amonts of online health information can heighten fear and anxiety. This increased anxiety may lead to higher utilization of medical services, adversely affecting individuals' perceptions of their health.

## 1 Introduction

The share of individuals who are turning to the Internet to obtain information about health has risen in the vast majority of European countries in recent decades, due to the widespread adoption of smartphones, tablets, and laptops. According to the Eurostat, around 52% of individuals searched for health-related information and symptoms online in 2022 and this proportion is still growing (Eurostat, 2022). Despite the Internet being an important and rapidly evolving source of health-related information, with negligible monetary and opportunity (time) costs (Bundorf et al., 2006; Costa-Font et al., 2009; Suenaga and Vicente, 2022), concerns about the quality of information available on the Internet and the ability of individuals to assess and understand its credibility and content are raising questions about the implications of expansions in its use (Suziedelyte, 2021; Chen and Liu, 2022).

Even hypothesizing that all the relevant medical information is available online, individuals do not have complete information about their own health conditions and may have limited abilities by which to utilize online information in such a way as to efficiently adopt health-improving medical decisions independently of their physicians (Arrow, 1963; Dwyer and Liu, 2013). This means that doctor-patient relationships cannot be replaced by patients self-diagnosing and medicating based on what they have found on the Internet, at least in principle. However, online information seeking behavior (henceforth e-HISB) might affect the likelihood of visiting a health professional as well as the frequency of visits and, ultimately, individuals’ health status.

There are two potential and contrasting hypothesis regarding the effects of e-HISB on physician visits according to Lee (2008): (i) online information seeking, by responding to patients’ needs for health information, may negatively affect the likelihood of visiting health professionals and the frequency of visits; (ii) conversely, e-HISB might increase their health concerns and consequently their demand for physician visits and other medical services by making individuals more acutely aware about their health conditions.

Despite e-HISB having become a global trend, only a few empirical investigations on how health information seeking from the Internet affects healthcare access and individuals’ health currently exist. Suziedelyte (2012) has investigated whether the health information that people obtain from the Internet affects their demand for healthcare using data from the U.S. Health Information National Trends Survey (2003–07). In the estimation model, she considered the endogeneity of Internet health information seeking in the demand for healthcare access equation and used information on U.S. states’ right-of-way regulations in order to construct an instrument for e-HISB. Her paper's findings suggest that Internet health information seeking has a positive effect on the demand for healthcare. According to her results, e-HISB makes patients more concerned about their health compared to non-seekers. Greater health awareness, in turn, drives e-health information seekers to increase their number of health professional visits.

Suenaga and Vicente (2022) examined the relationship between e-HISB and the demand for physician services, using data collected from the 2014 Eurobarometer survey on European citizens’ digital health literacy. Their analysis distinguished individuals seeking health information exclusively from offline sources from those seeking both online and offline sources. They used an extended sample selection model that addresses both the sample selection issue created by the survey design (i.e., the Eurobarometer survey collected data on offline health information searches only for those individuals who never sought health information online) and the endogeneity of health information seeking variables in the healthcare demand equation. The empirical analysis revealed that the demand for physician services is positively associated with offline health information seeking only and not with e-HISB, in contrast with previous findings by Suziedelyte (2012).

Hone et al. (2016), tried to understand whether e-HISB affects the likelihood of bad self-rated health with a logit regression model by using data collected from the 2014 Eurobarometer survey on European citizens’ digital health literacy. They distinguished between online seeking for general health and online seeking for disease-specific information. Their results show that searching for general information is less likely to be associated with self-reported bad health, whereas searching for disease-specific information increases the likelihood of self-reported bad health. However, the authors did not control for the e-HISB's endogeneity in the health equation and the related problems of reverse causality.

The previous literature failed to take the fact that e-HISB, individuals’ health and healthcare access might be determined simultaneously into account. For instance, individuals striving to deal with health challenges, such as an illness diagnosis or chronic disease management, tend to be much more motivated in e-HISBs (see for instance Ayers et al., 2007; Weaver et al., 2010): their health status may determine the demand for information to learn about a health or about illness-related concerns; e-HISB, as stated above, may affect (negatively or positively) the demand for healthcare services that, in turn, may influence individuals health status. Moreover, e-HISB is likely to be correlated to other variables that can also affect individuals’ demand for health and healthcare. Individuals who are more efficient producers of health, such as those who are highly educated and who have a higher level of health literacy, for instance, also have a greater ability to find and to act upon online health information, but are also simultaneously more likely to have a greater demand for health and healthcare services (Bundorf et al., 2006; Costa-Font et al., 2009).

The above discussion suggests that a step toward a complete understanding of the effects described requires a complex model that considers the simultaneous relationships between e-HISB, healthcare access, and an individual's health status. As such, we used a simultaneous equation model for binary variables; specifically, we constructed a joint model of e-HISB, healthcare access, and an individual's health status that considers individual's unobserved characteristics that are likely to be correlated with health information seeking, health status, and healthcare utilization.

In this study, we specifically focused on adults aged 50 and over. Although older adults show lower rates of Internet adoption, when compared to younger adults, online health information seeking is becoming increasingly common among them and this trend has accelerated as a result of the COVID-19 pandemic (Lee and Jang, 2022; Symeonaki et al., 2022). Health deteriorates with age, so older adults may be more motivated to seek health-related information in order to cope with uncertainty, to stay informed about preventing diseases, and to look for others with similar health concerns; at the same time, however, older adults might be more reluctant to depart from traditional paternalistic models of healthcare due to their limited proficiency with both computer usage and the Internet (Bundorf et al., 2006; Mesch et al., 2012).

We used data collected in the second SHARE Corona Survey, and supplemented them with data from the previous 8th wave of SHARE in order to assess both the potential merits and shortcomings of seeking health information online and how doing so may affect older adults healthcare access and health status. The second wave of the SHARE Corona Survey contains questions related to Internet access and the types of digital services used since the COVID outbreak (such as online banking, paying bills, or taxes, buying or selling goods, etc.) including questions concerning searches for information on health-related issues.

Individuals strongly relied on the Internet to stay informed during the COVID-19 pandemic outbreak and digital engagement grew in importance, especially among older adults because of lockdown mandates and social isolation (Suh et al., 2022); hence, COVID-19 serves as an exogenous source of variation. At the same time, the COVID-19 outbreak was characterized by the so-called *infodemic* phenomenon or an overabundance of health information available from a variety of (not always official or objective in nature) digital platforms that served to overwhelm the average person (WHO, 2020). We exploited the advantage of the SHARE Corona Survey in order to contribute to the understanding of e-HISB during exceptional times, such as the COVID-19 outbreak.

Our findings suggest that e-HISB can offer benefits by empowering individuals to enhance their understanding of health concerns. This, in turn, enables better health condition management. However, it may also lead to a chain reaction: navigating and evaluating the vast online health information and sources can heighten fear and anxiety. Consequently, increased anxiety may drive higher utilization of medical services, negatively impacting individuals' health perceptions.

The rest of the paper is organized as follows. Section 2 describes the data and variables used in this study and the empirical strategy deployed, including the estimation method. The results are discussed in Section 3. Finally, Section 4 concludes the paper.

## 2 Data and methods

### 2.1 Data

This study makes use of individual-level data drawn from the second SHARE Corona Survey. The first SHARE Corona Survey was implemented as a quick response within the SHARE study in order to understand the COVID-19 pandemic's effects. The interviews took place between June and September 2020 via a Computer Assisted Telephone Interview (CATI), partly to collect a set of basic information as in the regular SHARE questionnaire and partly to elicit information on life circumstances amidst COVID-19. Respondents who participated in the first SHARE Corona Survey were interviewed again and participated in the second SHARE Corona Survey from June to August 2021, which contained questions on their use of the Internet, including its use for information about matters pertaining to health. In addition to the second SHARE Corona Survey dataset, we use data from the regular 8th wave of SHARE, which collected information on the health, demographic, and socioeconomic status of respondents aged 50 years and over. The interviews took place between October 2019 and March 2020.

The final sample consisted of 13.829 observations across 18 European countries after conditioning for having no missing values on any dependent variable and/or covariate, namely: Austria, Belgium, Bulgaria, Croatia, Cyprus, Czech Republic, Denmark, Estonia, France, Italy, Latvia, Lithuania, Netherlands, Romania, Slovakia, Slovenia, Spain, and Sweden. The sample was restricted to exclude respondents living in European countries for which sub-national geographies were not available since our identification strategy is based on information collected from the 2020 Eurostat survey on the use of Information and Communication Technologies (ICT) in households and by individuals and the 2020 Eurostat data on the density of physicians at the Nomenclature of territorial units for statistics (NUTS) 2 level (see SubSection 2.5).^1^

### 2.2 Outcome variables

We identified three classes of dependent variables for the empirical model: e-HISB, healthcare access, and individuals’ general health status. e-HISB was defined as a binary indicator of whether respondents had looked for information on health-related issues on the Internet since the COVID-19 outbreak. According to their answer, they were classified as either e-HI seekers or as non-e-HI seekers.^2^

Concerning healthcare access, we created a binary variable indicating whether respondents went to a doctor's office or a medical facility in the last twelve months prior to the interview as a measure of health professional visits access.

We used self-assessed health (SAH) as a measure of an individual's health status. SAH is supported by literature that shows a strong predictive relationship between people's self-rating of their health and mortality or morbidity (Idler and Benyamini, 1997). Moreover, the self-assessed health measurement correlates strongly with more complex health indices, such as functional ability or indicators derived from health service use (Unden and Elofosson, 2006). The following standard self-assessed health status question was asked: ‘Would you say that in general your health is: 1. Excellent, 2. Very good, 3. Good, 4. Fair, 5. Poor?” We categorized the responses with multiple health status options into a binary indicator, assigning a value of 1 if individuals reported their health as fair or poor, and 0 otherwise (i.e., excellent, very good, or good). This approach was chosen as the responses could not be simply scored on a numerical scale (e.g., 1, 2, 3, 4, 5) due to the non-equidistant nature of the true scale between categories, as highlighted in previous literature (O'Donnell et al., 2008; Di Novi, 2010, 2013).^3^

All of the outcome variables were constructed according to the information included in the second SHARE Corona Survey.

**Fig. 1:**
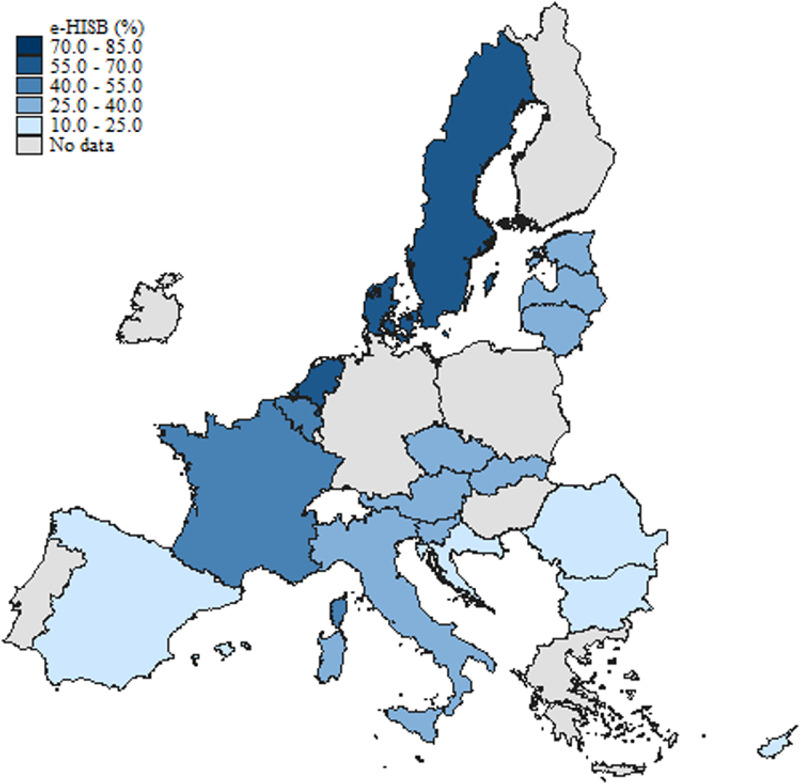
Distribution of e-HISB across European Countries included in the sample.

**Fig. 2:**
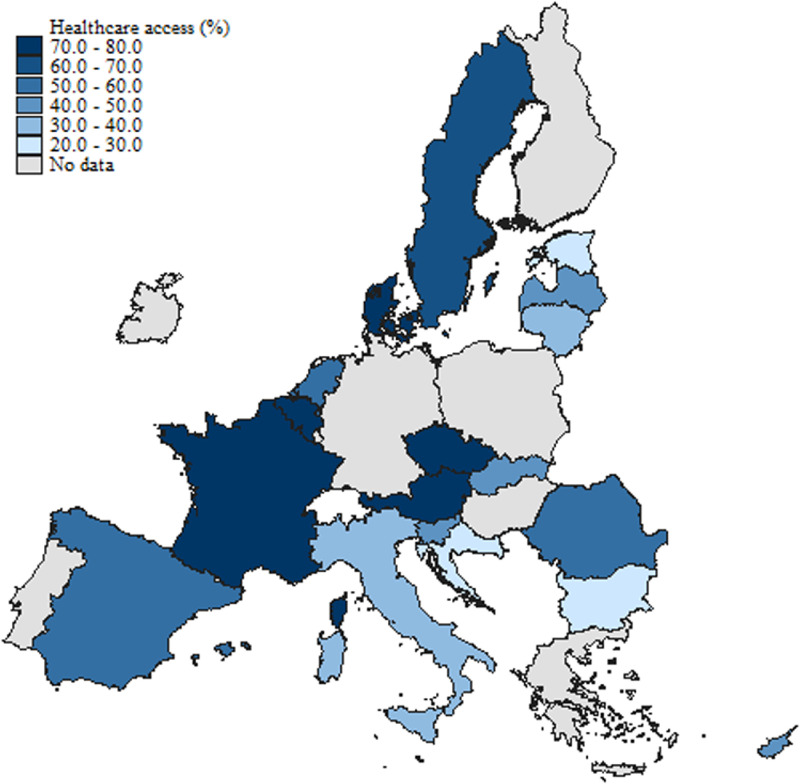
Distribution of health care access across European Countries included in the sample.

**Fig. 3:**
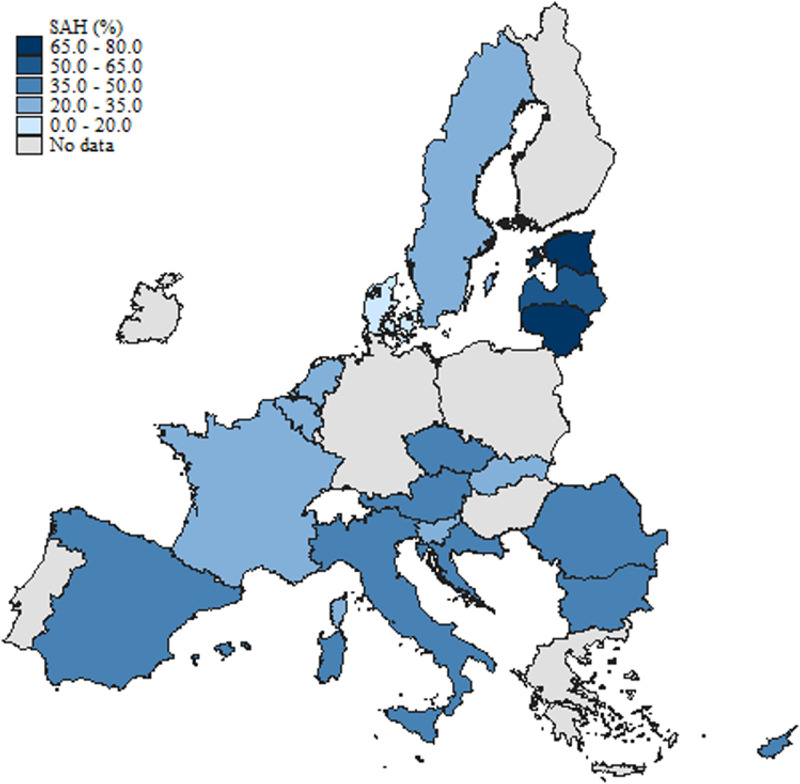
Distribution of poor SAH across European Countries included in the sample.

Figs. 1, 2, and 3 illustrate the geographical distribution of individuals seeking e-HISB, access to healthcare facilities, and occurrences of poor health across European countries included in the sample over the specified period (June-August 2021). The color intensity on the maps corresponds to the proportion of e-HI seekers, accessibility to health professional visits, and individuals experiencing poor health, respectively.

From Fig. 1, it is evident that the highest percentages of e-HI seekers are concentrated in Northern Europe. The color intensity on the map gradually diminishes when moving towards the South and East of Europe. This figure is in line with the evidence emerging from the *EU survey on the use of ICT in households and by individuals* (2021), where the highest shares of people seeking health information online for private purposes were recorded in the Netherlands and Denmark and the lowest in Bulgaria and Romania.^4^ Figs. 2 and 3, depicting access to health professional visits and instances of poor health respectively, reveal some, although not remarkable, differences across regions in terms of healthcare accessibility and health status. Shares of individuals with adverse health conditions are relatively higher among Southern and South-Eastern European countries, together with some Northern, post-socialist member states (Estonia, Latvia, Lithuania). The latter countries record significantly lower health status among the population compared to the other Northern and Western European countries. According to the *State of Health in the EU* report for 2021 and 2023 (State of Health in the EU, 2022, State of Health in the EU, 2023), the life expectancy at birth in these countries is still significantly lower than the EU average, with the largest gender gap among all EU countries. At the same time, they register the largest inequalities in self-rated health by income level, highest incidence of chronic conditions in the population, and largest obesity rates.^5^

Interestingly, higher shares of poor health correspond, up to some extent, to relatively lower healthcare accessibility. On the other side, countries with a lower incidence of adverse health in the population are also those with a higher intensity of healthcare utilization (Fig. 2). This is not surprising evidence, since lower healthcare accessibility may reflect lower overall health spending at the country level, both in per capita terms and as a share of GDP (State of Health in the EU, 2023). Indeed, high-spending countries like Sweden, Denmark, Austria, Belgium, and France have a high number of doctors (and/or nurses) compared to the EU average and, at the same time, relatively higher healthcare utilization. At the same time, countries like Estonia, Latvia, Lithuania, and Croatia register lower healthcare spending and professional visits (State of Health in the EU, 2023).

### 2.3 Explanatory variables

In our model, we controlled for a rich set of individuals’ demographic and socioeconomic characteristics, general health literacy, computer skills, and health conditions collected from the 8th wave of SHARE. For demographics, we included the respondent's sex, age, family size, geographic location (rural vs urban area), and a dummy variable for the region of residence. We incorporated controls for territorial confounding factors by utilizing macro-area controls corresponding to NUTS1 areas (i.e., major socioeconomic regions with a population ranging from 3 to 7 million people). We opted to include residence regions at the NUTS 1 level, as opposed to NUTS 2, in our controls to mitigate potential collinearity issues with the instruments used for model identification, while the choice of NUTS 1 over country dummies reflects our belief that this geographical subdivision more effectively captures the socioeconomic differences between regions.

For socioeconomic characteristics, we included individuals level of education, marital status, occupation, and income. The International standard classification of education (Isced) was used to classify the education variable. Three levels of education were considered: (1) low education (no educational certificates or primary school certificates or lower secondary education) as a reference category; (2) medium education (upper secondary education or high school graduation); and (3) high education (university degree or postgraduate). Marital status was categorized as ‘living with a spouse or a partner in the same household’ vs ‘living as single’ (reference category). Occupations were categorized into three groups: employed, retired, and other occupational status (namely unemployed, sick or disabled, homemakers, or other) as a reference category. Income information is based on total annual household income and was obtained by adding up its different components assessed in the questionnaire after deductions for income tax and social or national insurance contributions. It mainly comprises labor income, public pensions, and income from assets. Income was split into quartiles, with the lowest one as a reference category.

Suffering from health conditions is one of the most common reasons for accessing healthcare services, but also for gaining knowledge regarding health on the Internet (Rice, 2006). In our model, we controlled for suffering from a chronic disease (high blood pressure; high blood cholesterol; stroke; diabetes; chronic lung disease; asthma; arthritis, osteoporosis; cancer; peptic ulcer; Parkinson's disease; cataracts; hip fracture; or other conditions). Specifically, we created a dummy variable that takes value 1 if respondents reported that they suffer from at least one chronic condition. We also included an indicator in the model of pre-existing general health conditions that were identified by using the SAH dummy indicator (fair and poor vs excellent, very good, and good) from the 8th wave of SHARE.

Health literacy and computer skills have seen consistently reported as strong predictors for online health information seeking (Arnold et al., 2009; Kim, 2015). Moreover, health literacy can influence not only individuals’ e-HISB, but also its associated demand for health and healthcare services according to the previous literature (see, among others, Sørensen et al., 2012; Ilic et al., 2022). Indeed, health literacy affects an individuals’ ability to “*access, understand, appraise, and apply health information*” to what concerns health behaviors, health care access, and ultimately health outcomes (Sørensen et al., 2012, page 3). Hence, the model also included indicators of respondents' computer skills and general health literacy. Concerning computer skills, respondents were asked: “How would you rate your computer skill? Would you say they are …”. A five-point scale was used for the response, ranging from poor to excellent. An additional category was “I never used a computer”. We then derived a binary indicator that takes value 1 when respondents have at least good computer skills (i.e., when they reported 1. Excellent, 2. Very good, 3. Good) and 0 otherwise (4. Fair, 5. Poor, 6. I never used a computer”) (Cavapozzi and Dal Bianco, 2022). General health literacy was measured by using the single-item literacy screener (SILS), which was designed to identify adults in need of help with written or printed health material. Respondents were asked: “How often do you need to have someone help you when you read instructions, pamphlets or other written material from your doctor or pharmacy?” with answering options: 1. Never, 2. Rarely, 3. Sometimes, 4. Often and 5. Always. Once again, we constructed a dummy variable with a value of 1 if respondents reported “Never” and 0 otherwise (“Rarely”, “Sometimes”, “Often” and “Always”). Responses “Never” were selected to represent a good level of health literacy.

We observed individuals’ e-HISB, health, and healthcare access during an exceptional time, namely the COVID-19 pandemic. The COVID-19 context was characterized by uncertainty and a strong need for information about the pandemic's evolution, the risks associated with coronavirus exposure, the community-level policies, and restrictions. The local virus spread might also have been a key factor in determining e-HISB, healthcare access, and individuals’ health (especially in terms of psychological distress). Therefore, the model considered a variable related to the COVID-19 experience and to the spread of COVID-19 among respondents’ contacts. This dummy indicator has a value of 1 if a respondent or anyone close to a respondent had suffered from the Coronavirus or had been hospitalized due to the infection or if anyone close to a respondent died after having become infected by the Coronavirus, and 0 otherwise.

Finally, following Di Novi et al. (2023), we also included the COVID-19 Government Response Stringency Index (SI) from the Oxford Coronavirus Government Response Tracker (OxCGRT) in the model (Hale et al., 2021).^6^ This index captures the day-to-day variation in the containment and closure policies adopted by national governments worldwide to tackle the pandemic. The index scores between 0 and 100, with a higher score indicating a more stringent response. The SI relies on the following measures: the closure of schools and universities, workplaces, and public events; limitations on gatherings; suspension of public transport services; directives to 'shelter-in-place' or stay confined at home; restrictions on internal movement between cities/regions; limitations on international travel; and the implementation of public information campaigns.

It was possible to know each participant's interview month from the SHARE Corona Survey questionnaire. The average value of the SI was computed over the month of the interview in the respondent's country of residence. This value was then compared with the value of the SI in the same country by March 12, 2020 (the day after WHO declared COVID-19 as a pandemic) to capture the potential mitigation/tightening in the COVID-19 restrictions from the beginning of the pandemic; this might have influenced an individual's healthcare access, e-HISB, and their health (especially in terms of psychological distress). We then constructed a binary variable that takes the value of 1 if the stringency index has been declining from the beginning of the COVID-19 pandemic to the period of observation at the country level and 0 otherwise.

Table 1 sets out a full description of the variables used in the model.

**Table 1:** Variables Description and Data Source.

e-HISB:	1 if respondent uses internet to look for health information (data source: second SHARE Corona Survey)
Broadband	percentage of individuals, at NUTS2, that have broad band access(data source: 2020 Eurostat survey on the use of ITC)
Health professional visits	1 if respondent declares to have seen doctor/medical facility other than hospital since last interview (data source: second SHARE Corona Survey)
N. of physicians	n. of physicians/1.000.000 inhabitants, at NUTS2 level (data source: 2020 Eurostat data on the density of physicians)
SAH	1 if respondent suffered from fair-poor health (data source: second SHARE Corona Survey)
SAH_t-1_	1 if respondent suffered from fair-poor health (data source: 8th wave of SHARE)
Chronic condition	1 if respondent suffered from at least one chronic disease in 2019/2020 (data source: 8th wave of SHARE)
Rural	1 if respondent lives in rural area (data source: 8th wave of SHARE)
Health literacy	1 if respondent declares no need to help with reading health information (data source: 8th wave of SHARE)
Female	1 if respondent is female (data source: 8th wave of SHARE)
Household size	n. of individuals within the household (data source: 8th wave of SHARE)
Age	age as continuous variable (data source: 8th wave of SHARE)
Local spread covid	1 if respondent or anyone close to a respondent had suffered from the Coronavirus or was hospitalized due to the infection or anyone close to a respondent died after being affected by the Coronavirus (data source: second SHARE Corona Survey)
Stringency index (decline)	1 if the stringency index in the country of residence has been declining from the beginning of the COVID-19 pandemic to the month of the interview (data source: Oxford Coronavirus Government Response Tracker - OxCGRT)
High pcskill	1 if respondent has at least good computer skills, and 0 otherwise (data source: 8th wave of SHARE)
Marital status	1 if the individual is lives with, 0 otherwise (data source: 8th wave of SHARE)
Retired	1 if respondent is retired (data source: 8th wave of SHARE)
Employed	1 if respondent is employed (data source: 8th wave of SHARE)
Other occupations	1 if respondent l is unemployed, sick or disabled, home maker or is doing other (data source: 8th wave of SHARE)
Low educ	1 if respondent reported low education level (data source: 8th wave of SHARE)
Medium educ	1 if respondent reported medium level of education (data source: 8th wave of SHARE)
High educ	1 if respondent reported high education level (data source: 8th wave of SHARE)
Quartiles	dummies for income quartiles (data source: 8th wave of SHARE)
NUTS 1	dummies for Nomenclature of Territorial Units for Statistics (NUTS) 1 level

### 2.4 Empirical strategy

Identifying a causal relation between e-HISB, health professional visits, and health may be complicated by the presence of endogeneity, as stated previously. Indeed, e-HISB may be correlated to either unobserved health characteristics or to unobserved preferences that are likely to influence the demand for health and healthcare services. As such, we estimated the model using a recursive multivariate probit design.^7^ The multivariate probit model's recursive structure builds on two structural-form equations that determine the probability of poor health status and healthcare access, and one reduced-form equation for the potentially endogenous dummy variable measuring e-HISB. In the equation for health professional visits, the e-HISB indicator is included as an explanatory variable. Its inclusion allowed us to test Lee's hypothesis (2008) — examining whether e-HISB could either decrease the likelihood of visiting health professionals, acting as a substitute, or increase the demand for medical services by raising individuals' awareness of their health conditions. e-HISB and access to physicians are included as regressors in the structural equation for health.

We constructed and estimated a system of three equations with one reduced-form equation (*e-HISB_i*) and two structural equations (*Health Status_i* and *Healthcare Access_i*). Thus:

(1) $$\begin{array}{c} \text{Health}\;\text{Statu}s_{i}=\delta_{1}y\text{Healthcare}\;\text{Acces}s_{i}+\delta_{2}e-\text{HIS}B_{i}+{\alpha^{\prime }}_{1}Z_{1i}+\varepsilon_{1i}\\ \text{Healthcare}\;\text{Acces}s_{i}=\gamma_{2}e-\text{HIS}B_{I}+{\alpha^{\prime }}_{2}Z_{2i}+\varepsilon_{2i}\\ e-\text{HIS}B_{i}={\alpha^{\prime }}_{3}Z_{3i}+\varepsilon_{3i} \end{array}$$

where ***Z****_hi_* (with *h* = 1, 2, 3) are vectors of exogenous variables, α_h_ are parameter vectors, and $\delta_{o}$ (with *o* = 1, 2) and $\gamma_{2}$ are scalar parameters. The error terms distributed as multivariate normal are $\varepsilon_{hi}$, each with a mean 0 and variance covariance matrix Σ. Σ has values of 1 on the leading diagonal and correlations $\rho_{jk}=\rho_{kj}$ on the off-diagonal elements (where $\rho_{jk}$ is the covariance between the error terms of equation *j* and *k*).

The exogeneity condition is stated in terms of the correlation coefficients in the setting mentioned previously, which can be interpreted as the correlation between the different equations’ unobservable explanatory variables. All equations in system (1) can only be estimated separately as single probit models in the case of independent error terms (i.e., the coefficient $\rho_{jk}\;$is not significantly different from zero).

Conventionally, the identification of a recursive multivariate probit model has been based on exclusion restrictions in order to obtain a more robust identification of the parameters. According to Maddala (1983), at least one of exogenous variables of the e-HISB and physicians access equations (i.e., in the vectors $z_{2i}$and $z_{3i}$) are not included in the health equation as explanatory variables. However, more recent work by Wilde (2000) shows that identification is achieved even if the same regressors appear in all equations, providing that there is sufficient variation in the data (i.e., providing that each equation contains at least one varying exogenous regressor). However, this result is valid in the context of multivariate normal distribution and, in the absence of additional instruments, identification strongly relies upon functional form—i.e., the normality of the stochastic disturbances, commonly referred to as identification by functional form (Li et al., 2019). It is, therefore, common practice to impose exclusion restrictions in order to improve the identification of the causal parameters$\;\delta_{1},\;\delta_{2}$ and $\gamma_{2}$. The instruments are discussed in detail in SubSection 2.5.

### 2.5 Exclusion restrictions

This subsection describes the exclusion restrictions that we adopted for both e-HISB and healthcare access equations.

#### 2.5.1 e-HISB equation

We exploited the heterogeneity in regional NUTS-2 on broadband coverage in order to deal with the potential endogeneity of e-HISB. Specifically, we used data from the Eurostat database on Information and Communication Technology (ICT) usage in households and by individuals and measure broadband internet diffusion with the variable that refers to the percentage of households with broadband internet access (*isoc_r_broad_h*).

In recent years, broadband infrastructures and network speeds across European countries have improved substantially. The majority of European countries now have at least 80 percent of their households equipped with broadband access, enabling high-speed connections (> 30 Mbps) and very high-speed connections (> 100 Mbps). Bulgaria and Italy, however, exhibit slightly lower levels, ranging between 82 and 85 percent, respectively.

We assumed that the high-speed connection increases the frequency of Internet use and the engagement with Internet activities, thereby facilitating information searches; moreover, the high-speed connection might also mean that individuals can access more content in a given amount of time. Hence, we expected that we might observe an association between the high-speed connection and e-HISB because of the enhanced internet access enabled by faster broadband speeds (McDool et al., 2020).

#### 2.5.2 Health professional visits equation

In order to address the potential endogeneity of health professional visits’ binary indicator in the health equation, we included, in the vector $Z_{2i}$, an indicator of healthcare supply at the regional level (NUTS-2); namely, the number of medical doctors, including generalists and specialist medical practitioners per 1000,000 inhabitants provided by Eurostat.

We expected that the number of doctors and their geographic distribution might influence the likelihood of accessing a health professional in normal circumstances and even more so during exceptional times, such as during the COVID-19 pandemic.

## 3 Results

Table 2 shows a simple descriptive analysis that presents sample means and standard deviations for the variables used in the model. About 36% of the study sample (60% female; mean age: 71 years) used the Internet during the COVID-19 outbreak to search for health information. We might note that the prevalence of bad health, based on SAH, increased from around 40% at the time of wave 8 to 42% by the time of the second COVID Survey. About 50% of respondents went to a doctor's office or a medical facility in the previous twelve months prior to the interview.

**Table 2:** Descriptive Statistics.

Panel A: Full sample			
Variable	Mean	Std. Dev.	N
e-HISB	0.362	0.481	13,829
Broadband	89.783	3.578	13,829
Health professional visits	0.501	0.500	13,829
N. of physicians	36.387	6.857	13,829
SAH	0.424	0.494	13,829
SAH_t-1_	0.408	0.491	13,829
Chronic conditions	0.744	0.436	13,829
Rural	0.361	0.480	13,829
Health literacy	0.731	0.443	13,829
High pcskill	0.323	0.468	13,829
Female	0.602	0.489	13,829
Household size	2.062	0.980	13,829
Age	71.206	9.108	13,829
Local spread covid	0.402	0.490	13,829
Stringency index (decline)	0.302	0.459	13,829
Marital status	0.675	0.469	13,829
Retired	0.691	0.462	13,829
Employed	0.199	0.399	13,829
Other occupations	0.098	0.298	13,829
Low educ	0.322	0.467	13,829
Medium educ	0.457	0.498	13,829
High educ	0.221	0.415	13,829
1°quartile	0.277	0.447	13,829
2°quartile	0.279	0.448	13,829
3°quartile	0.241	0.428	13,829
4°quartile	0.202	0.402	13,829

Panel B: Non -HI seekers.			
Variables	Mean	Std. Dev.	N
Broadband	89.593	3.657	8812
Health professional visits	0.466	0.499	8812
N. of physicians	361.820	69.055	8812
SAH	0.487	0.500	8812
SAH_t-1_	0.471	0.499	8812
Chronic conditions	0.777	0.416	8812
Rural	0.402	0.490	8812
Health literacy	0.664	0.472	8812
High pcskill	0.179	0.383	8812
Female	0.603	0.489	8812
Household size	2.035	1.021	8812
Age	73.422	9.155	8812
Local spread covid	0.357	0.479	8812
Stringency index (decline)	0.328	0.470	8812
Marital status	0.634	0.482	8812
Retired	0.758	0.428	8812
Employed	0.121	0.326	8812
Other occupations	0.108	0.311	8812
Low educ	0.417	0.493	8812
Medium educ	0.444	0.497	8812
High educ	0.138	0.345	8812
1°quartile	0.347	0.476	8812
2°quartile	0.304	0.460	8812
3°quartile	0.209	0.406	8812
4°quartile	0.140	0.347	8812

Panel C: e-HI seekers			
Variables	Mean	Std. Dev.	Obs
Broadband	90.118	3.410	5017
Health professional visits	0.563	0.496	5017
N. of physicians	367.499	67.574	5017
SAH	0.315	0.464	5017
SAH_t-1_	0.296	0.457	5017
Chronic conditions	0.687	0.464	5017
Rural	0.287	0.452	5017
Health literacy	0.849	0.358	5017
High pcskill	0.576	0.494	5017
Female	0.602	0.490	5017
Household size	2.111	0.900	5017
Age	67.302	7.587	5017
Local spread covid	0.481	0.500	5017
Stringency index (decline)	0.257	0.437	5017
Marital status	0.746	0.435	5017
Retired	0.572	0.495	5017
Employed	0.338	0.473	5017
Other occupations	0.082	0.274	5017
Low educ	0.154	0.361	5017
Medium educ	0.478	0.500	5017
High educ	0.368	0.482	5017
1°quartile	0.155	0.362	5017
2°quartile	0.234	0.424	5017
3°quartile	0.299	0.458	5017
4°quartile	0.311	0.463	5017

About 31% of e-HI seekers reported suffering from fair or poor health against about 49% of non-seekers. The proportion of those who have accessed health professionals and medical facilities is higher among e-HI seekers: about 56% against 47% among non-seekers. e-HI seekers are younger (mean age of e-HI seekers 67 years against 73 years of non-seekers) and have a higher level of health literacy and computer skills compared with non-seekers: about 85% of e-HI seekers reported having good health literacy against 66% of non-seekers; 55% of e-HI seekers also reported having higher computer skills against 18% of non-seekers respectively.

Table 3 shows the estimated marginal effects for the structural equations for bad health status and health professional visits and medical facility access and the reduced-form equation for an individual's e-HISB. In all estimations, the standard errors are clustered to account for the utilization of NUTS 2 level variables as instruments for Internet health information seeking and health professional visits, respectively, following the approach outlined by Wooldridge (2003).

**Table 3:** Multivariate probit model results: marginal effects.

	e-HISB	Health professional visits	SAH
SAH_t-1_	−0.026	0.041***	0.350***
	(0.037)	(0.031)	(0.040)
Chronic conditions	0.034***	0.132***	0.110***
	(0.034)	(0.029)	(0.037)
Rural	−0.055***	−0.001	0.018*
	(0.049)	(0.041)	(0.028)
Health literacy	0.058***	0.021	−0.080***
	(0.069)	(0.040)	(0.038)
High pcskill	0.192***	−0.001	−0.036***
	(0.019)	(0.016)	(0.015)
Female	0.023***	0.016**	−0.003
	(0.031)	(0.023)	(0.028)
Household size	−0.016*	−0.009*	0.002
	(0.022)	(0.015)	(0.018)
Age	−0.013***	−0.001	0.003***
	(0.002)	(0.002)	(0.002)
Local spread covid	0.052***	0.039***	0.020***
	(0.031)	(0.030)	(0.028)
Stringency index (decline)	0.012*	0.033***	−0.009
	(0.033)	(0.034)	(0.031)
Marital status	0.038***	0.008	−0.003
	(0.038)	(0.035)	(0.046)
Retired	0.047***	0.040***	−0.013
	(0.049)	(0.042)	(0.043)
Employed	0.038***	0.008	−0.043**
	(0.046)	(0.046)	(0.058)
Medium educ	0.097***	0.012	−0.025**
	(0.036)	(0.032)	(0.033)
High educ	0.205***	0.045***	−0.049***
	(0.041)	(0.041)	(0.039)
2°quartile	0.030**	0.024**	−0.021
	(0.054)	(0.034)	(0.049)
3°quartile	0.072***	0.027*	−0.030**
	(0.055)	(0.042)	(0.048)
4°quartile	0.077***	0.012	−0.033
	(0.055)	(0.057)	(0.064)
Broadband	0.017***		
	(0.008)		
N. of physicians		0.004***	
		(0.001)	
e-HISB		0.061***	−0.017***
		(0.052)	(0.062)
Health professional visits			0.057***
			(0.072)
NUTS 1 dummies	yes	Yes	yes
N	13,829	13,829	13,829

Notes: Significance levels: * *p* < 0.1, ** *p* < 0.05, *** *p* < 0.01. All the reported coefficients are average marginal effects. Standard errors are clustered at NUTS2 level.

With specific reference to the reduced-form equation, our findings show that the indicator for broadband internet diffusion has a positive and significant effect on an individual's e-HISB, as expected: it increases the probability of accessing the Internet for searching health information by about 1.7%. Indeed, as stated previously, high-speed connections increase the frequency of Internet use by facilitating health information searches by reducing the opportunity time cost of accessing information on the Internet.

While the dummy indicator of pre-existing general health conditions, based on SAH, does not influence e-HISB, having been diagnosed with a chronic health condition increases the probability of being an e-HI seeker of about 3.4%. This result is consistent with the findings of previous studies that reported that one of the main reasons to go online to search for health information was having been diagnosed with a specific medical condition (see Bundorf et al., 2006; Rice, 2006; McMullan, 2006).^8^

According to our results, individuals responded to the local spread of the coronavirus by searching for health information online (with a marginal effect of about 5.2%). The uncertainty of the nature of the disease and the method of transmission and treatment might have led individuals to seek out health-related information on COVID-19, coronavirus symptoms, and its treatment.^9^ e-HISB is negatively affected by age and living in a rural area, while it is positively affected by being female, married, highly educated, with a higher level of income, with a good level of health literacy, and having computer skills as expected (which increased the probability of being an e-HI seeker from about 5.8% to 19.2% respectively).

With reference to the structural equation for the likelihood of visiting a health professional or accessing a medical facility (Column 2 in Table 3), our results show that an increasing number of medical doctors had a positive and significant effect on healthcare professionals access (+0.4%).

Respondents in poor health have a greater demand for health professionals than those in better health and were more likely to go to either a doctor's office or a medical facility as expected (i.e., self-reported health status increases the probability of accessing a healthcare professional by about 4.1% while suffering from at least one chronic condition raises this likelihood by about 13.2%). According to our results, the likelihood of visiting a health professional or of accessing a medical facility is also positively affected by being female and highly educated and by the mitigation in the COVID-19 restrictions from the beginning of the pandemic (+3.3%).^10^

Finally, the results of the empirical analysis show that searching for health information on the Internet, all other factors being equal, has a positive and statistically significant effect on an individual's demand for healthcare professionals and medical facility access with a marginal effect of about 6%: it is apparent that e-HI seekers demand more health care than non-seekers. These findings support the hypothesis that e-HISB, by increasing individuals' awareness of their health conditions, heightens their health concerns. Consequently, this heightened awareness may drive e-HI seekers to consult either a healthcare professional or a medical facility. Another potential scenario is that inaccurate online health information might lead to patients making incorrect self-diagnoses, prompting more frequent visits to the doctor (Lee, 2008; Suziedelyte, 2012). However, according to our results (see Column 3 in Table 3), visiting a health professional or accessing medical facilities increases the likelihood of perceiving poor health by about 5.7%. So, can searching for health information online increase the likelihood of accessing healthcare services and, at the same time, lead to a perception of a worse state of health? Firstly, it is essential to consider that the indicator of perceived health also captures significant psychological components such as negative mood and perceived vulnerability to illness. These factors appear to be significant contributors to self-assessed health independently of an individual's physical dimensions, such as physical symptoms and diseases. Psychological factors play a relatively major role in how “healthy” we feel compared to physical discomfort (see, for instance, Andersen and Lobel, 1995; Benyamini et al., 2000). A medical visit can increase an individual's perceived vulnerability to disease, inducing anxiety, particularly in cases involving suspected health issues. This anxiety may contribute to a negative perception of one's health status, potentially explaining the correlation between seeking health professionals or accessing medical facilities and reporting lower self-rated health (Culica et al., 2002; Labeit et al., 2013). It could be argued that seeking information on the internet has its benefits. On one hand, it can help individuals gain knowledge about their health concerns, enabling them to better manage their health conditions through comprehensive information on medical conditions, prevention strategies, and treatment options (Lambert and Loiselle, 2007). Indeed, when we focus on the direct impact of e-HISB on an individual's poor health status (see Table 3, column 3), we observe a negative effect. This means that engaging in online health information seeking is associated with a decreased likelihood of perceiving bad health, with a marginal effect of approximately −1.7%. On the other hand, however, e-HISB might trigger a sort of chain reaction: when individuals search online for information about their health, the challenge of navigating and evaluating the credibility of the vast amount of information and its sources may lead to heightened levels of fear and anxiety. The inability to comprehend the content of information or assess its reliability can amplify existing concerns and contribute to the emergence of new ones. This, in turn, could result in an increased utilization of medical services and a more negative perception of one's own health.^11^ Examining the estimated marginal effects with our model, our results indicate that the “net” effect of e-HISB tends to lean more towards the latter aspect, creating a sort of vicious circle. In this scenario, the patient, frightened by the news found online, is more likely to consult the doctor or seek additional healthcare services, leading to a subsequent increase in anxiety and negative repercussions on their perceived health.

Concerning the other variables included in the structural equation, our findings show that a good level of health literacy, higher computer skills, and a higher socioeconomic status in general were associated with a lower probability of reporting poor health that increases with higher age and previously existing poor health conditions.

As discussed previously, we constructed a simultaneous equation model for three binary variables. The multivariate probit estimation allowed us to test for unobserved heterogeneity that may characterize the relationship between e-HISB and an individual's healthcare access and health status. The unobserved heterogeneity is captured by the correlation between the error terms from the single equation models. Table 4 shows the full recursive model's correlation coefficients.

**Table 4:** Correlation coefficients between the disturbance of the e-HISB, health professional visits e and individuals’ health status equations.

	e-HISB	Health professional visits	SAH
e-HISB	1	0.008(0.028)	0.070*(0.029)
Health professional visits		1	−0.026(0.036)
SAH			1

Standard errors in parentheses.

Legend: * = 10% significance level.

The null hypothesis of exogeneity is rejected in only one case. According to our results, there exists a positive and statistically significant correlation between the disturbance of the e-HISB equation and the structural equation for individuals’ poor health status—i.e., e-HISB seems to be disease or illness oriented, indeed unobservable variables that increase the likelihood of bad health and also increase the probability of searching for health information online.

### 3.1 The role of age

In this section, we examined how our model's results can vary based on individuals' age. The health of individuals generally declines with age: older individuals are also more prone to experiencing chronic diseases. This may prompt them to seek health-related information online, driven by the need to manage uncertainty, stay informed about preventive measures against diseases and connect with peers facing similar health challenges. However, it is crucial to emphasize that older adults may be resistant to transitioning away from traditional healthcare models due to their limited proficiency in using computers and the Internet. This limitation has been documented in studies by Bundorf et al. (2006) and Mesch et al. (2012). These aspects could make the analysis by age groups more intriguing.

To examine the potential influence of interviewees' age on our analysis results, we divided the sample into two groups: respondents aged between 50 and 69 years and those aged 70 or older. In our sample, approximately 23% of individuals aged 70 and older reported using the Internet to find health-related information, compared to a higher percentage of 50% in the 50 to 69 age group. Regarding computer skills, around 21% of individuals aged 70 and older demonstrated a high level of proficiency, while this percentage was higher at 45% for younger adults aged between 50 and 69. Detailed summary statistics related to the two different age groups can be found in the Appendix (see Table A3).

Table 5 only presents the marginal effects for the variables of interest (the complete model is available from the authors).

**Table 5:** Multivariate probit model – marginal effects - by age groups.

Panel A: Individuals aged 50–69			
	e-HISB	Health Professional Visits	SAH
Broadband	0.015***		
	(0.011)		
N. of physicians		0.003*	
		(0.006)	
e-HISB		0.049***	−0.021**
		(0.097)	(0.085)
Health professional visits			0.072***
			(0.100)
N	6316	6316	6316

Panel B: Individuals aged 70 and older			
Broadband	0.044***		
	(0.018)		
N. of physicians		0.006***	
		(0.005)	
e-HISB		0.075**	−0.013
		(0.067)	(0.115)
Health professional visits			0.046*
			(0.115)
N	7513	7513	7513

Notes: Significance levels: * *p* < 0.1, ** *p* < 0.05, *** *p* < 0.01. All the reported coefficients are average marginal effects. We have included all the control variables as in the main specification. Standard errors are clustered at NUTS2 level.

Apparently, the aforementioned phenomenon, characterized as a sort of self-perpetuating cycle wherein individuals, having sought health-related information on the Internet, are more likely to consult a doctor—resulting in heightened stress that detrimentally influences their perceived health—primarily impacts the younger part of our sample, specifically those aged between 50 and 69. Interestingly, this effect is not observed in the over-70 age group. Within the younger segment of our sample, there is also a direct, negative, and statistically significant effect of e-HISB on poor self-perceived health, revealing a (albeit relatively smaller) beneficial effect of e-HISB on individual health status. Once again, however, when contrasted with its indirect impact mediated by increased healthcare access (described above), this yields an overall adverse net balance of e-HISB on individual perceived health.

## 4 Conclusions

Digitalization has impacted numerous sectors of the economy, but perhaps one of the most significant changes has occurred in the healthcare sector, where so-called e-health plays an increasingly prominent role. The COVID-19 pandemic has effectively accelerated this process, introducing new tools and innovative approaches that undoubtedly speed up access to healthcare services, the management of clinical information, and, ultimately, active patient involvement. However, this process has simultaneously created new challenges. One of the most noticeable aspects of the digitalization of the healthcare sector is the emergence of platforms and online health services that provide a fertile ground for e-HISB. These solutions, often accessible through mobiles, tablets, and computers, enable patients to access detailed health information that can be consulted at any time and from anywhere, but they can also influence their health-related decisions.

Our findings suggest that seeking information on the internet can yield potential benefits, as e-HISB empowers individuals to enhance their knowledge about health concerns and symptoms, enabling them to better manage their health conditions. However, e-HISB may also trigger a chain reaction: when individuals search online for health information, the challenge of navigating and evaluating the credibility of the vast amount of information and its sources may lead to heightened levels of fear and anxiety. Consequently, this increased anxiety could result in a higher utilization of medical services, which, in turn, may negatively impact individuals' perception of their health.

Our findings highlight the importance of policy interventions aimed at promoting the availability of accurate and reliable health information online. Health authorities could collaborate with digital platforms to ensure the presence of verified and approved content.

In an era where digitalization plays a fundamental role in health and healthcare services, it would also beadvisable to promote educational programs on digital health. These programs can equip individuals with the skills to critically and responsibly search for health information, thereby helping to alleviate anxiety stemming from misleading or unverified information.

## Funding

This research did not receive any specific grant from funding agencies in the public, commercial, or not-for-profit sectors.

## Declaration of competing interest

The authors declare that they have no known competing financial interests or personal relationships that could have appeared to influence the work reported in this paper.

## Figures and Tables

**Table A1:** Multivariate Probit Model - health deterioration* as a dependent variable.

	e-HISB	Health professional visit	SAH
			
Broadband	0.017***		
	(0.008)		
N. of physicians		0.004***	
		(0.001)	
e-HISB		0.060**	−0.006**
		(0.011)	(0.007)
Health professional visits			0.024**
			(0.008)
NUTS 1 dummies	yes	yes	yes
N	13,829	13,829	13,829

Notes: Significance levels: * *p* < 0.1, ** *p* < 0.05, *** *p* < 0.01. All the reported coefficients are average marginal effects. Standard errors are clustered at NUTS2 level.

*We carried out a sensitivity analysis re-running the model with a different construction for SAH: starting from the original variables on perceived health, measured before and during the pandemic, we created a dichotomous variable that takes the value of 1 in the case of reported health deterioration. By "deterioration," we mean a lower score in self-reported health. For example, a person who reported a perceived health rating of 1 (excellent) in the eighth wave of SHARE and declared a perceived health rating of 2 (very good) in 2021 would be recorded as someone who has experienced deterioration. This construction did not significantly affect the results: the multivariate probit coefficients with this new dependent variable were fairly similar to those presented in the paper.

**Table A2:** Correlation coefficients between the disturbance of the e-HISB, health professional visits e and health deterioration equations.

	e-HISB	Health professional visits	SAH
e-HISB	1	0.008 (0.028)	0.064*(0.034)
Health professional visits		1	−0.041(0.048)
SAH			1

Standard errors in parentheses.

Legend: * = 10% significance level.

**Table A3:** Descriptive statistics by age groups.

Panel A: age group 50–69			
Variables	Mean	Std. Dev.	N
e-HISB	0.508	0.499	6329
Broadband	89.49	3.632	6329
Health professional visits	0.488	0.499	6329
N. of physicians	35.942	6.931	6329
SAH	0.315	0.464	6329
SAH_t-1_	0.31	0.462	6329
Chronic conditions	0.627	0.483	6329
Rural	0.386	0.486	6329
Health literacy	0.794	0.404	6329
High pcskill	0.454	0.497	6329
Female	0.599	0.489	6329
Household size	2.299	1.046	6329
Age	63.06	4.199	6329
Local spread covid	0.441	0.496	6329
Stringency index (decline)	0.314	0.464	6329
Marital status	0.765	0.423	6329
Retired	0.42	0.493	6329
Employed	0.41	0.492	6329
Other occupations	0.154	0.361	6329
Low educ	0.237	0.425	6329
Medium educ	0.521	0.499	6329
High educ	0.24	0.427	6329
1°quartile	0.235	0.424	6329
2°quartile	0.25	0.433	6329
3°quartile	0.267	0.442	6329
4°quartile	0.247	0.431	6329

Panel B: group 70 and over			
Variables	Mean	Std. Dev.	N
e-HISB	0.239	0.426	7541
Broadband	90.02	0.511	7541
Health professional visits	0.511	0.499	7541
N. of physicians	36.76	6.772	7541
SAH	0.515	0.499	7541
SAH_t-1_	0.489	0.499	7541
Chronic conditions	0.842	0.364	7541
Rural	0.339	0.473	7541
Health literacy	0.678	0.467	7541
High pcskill	0.121	0.409	7541
Female	0.604	0.488	7541
Household size	1.862	0.871	7541
Age	78.03	5.952	7541
Local spread covid	0.369	0.482	7541
Stringency index (decline)	0.292	0.454	7541
Marital status	0.598	0.49	7541
Retired	0.917	0.275	7541
Employed	0.021	0.145	7541
Other occupations	0.052	0.223	7541
Low educ	0.393	0.488	7541
Medium educ	0.401	0.49	7541
High educ	0.204	0.403	7541
1°quartile	0.313	0.463	7541
2°quartile	0.302	0.459	7541
3°quartile	0.219	0.413	7541
4°quartile	0.164	0.37	7541

## Data Availability

Data will be made available on request.

## Appendix

Table A1, Table A2, Table A3

## Footnotes

1 The EU survey on the use of Information and Communication Technologies (ICT) in households and by individuals has been conducted every year since 2002 and collects harmonized and comparable data on households’ access to, and individuals’ use of, the Internet.

2 The question we referred to, contained in the SHARE Corona Survey questionnaire, is as follows: “Nowadays, many things can be done online, that is, via the Internet. Since the outbreak of Corona, have you used the Internet more often, about the same, less often, or not at all for the following online activities?” One of the responses included "Searching for information on health-related issues." We have then created a binary variable that takes the value of one if respondents answered that they have used the Internet "more often, about the same, less often" and zero otherwise ("or not at all"). This differentiation allowed us to distinguish between individuals actively seeking information on health-related issues and those who do not, irrespective of the frequency of their internet usage. Indeed, our objective was simply to distinguish between e-HI seekers or as non-e-HI seekers.

3 Some questions in health surveys require binary responses (yes or no). Based on these responses, samples can be divided into individuals experiencing health-related issues and those who are not. Other questions utilize ordered response options. SAH comprises categories like (i) excellent, (ii) very good, (iii) good, (iv) fair, or (v) poor. Simply assigning numerical scores, such as 1, 2, 3, 4, 5, is inadequate as perceived health, measured this way, cannot be truly considered a scale, as the distance between "excellent" and "very good" or "good" and "fair", for instance, are not identical. According to O'Donnell et al. (2008) and subsequent literature we dichotomized SAH into a binary variable that measures health as the proportion of individuals exhibiting a specific trait, such as those indicating their health as “less than good”. We carried out a sensitivity analysis re-running the model with a different construction for SAH: starting from the original variables on perceived health, measured before and during the pandemic, we created a dichotomous variable that takes the value of 1 in the case of reported health deterioration. By "deterioration" we mean a lower score in self-reported health. For example, a person who reported a perceived health rating of 1 (excellent) in the eighth wave of SHARE and declared a perceived health rating of 2 (very good) in 2021 would be recorded as someone who has experienced deterioration. This construction did not significantly affect the results: the multivariate probit coefficients with this new dependent variable were fairly similar to those presented in the paper. The results of the sensitivity analysis are included in the Appendix (see Tables A1 and A2).

4 For more info, see: https://ec.europa.eu/eurostat/web/products-eurostat-news/-/edn-20220406-1

5 For more info, see, for instance: https://eurohealthobservatory.who.int/news-room/news/item/13-12-2021-state-of-health-in-the-eu-2021-latest-evidence-on-29-countries-health-systems-performance

6 Free publicly-accessible data collected by the OxCGRT was used; it is available here: https://www.bsg.ox.ac.uk/research/research-projects/ covid-19-government-response-tracker.

7 A recursive model is a special case of a system of equations in which the endogenous variables are determined in a sequence. Thus, the right-hand side of the reduced-form equations for the endogenous variables include exogenous variables only. The right-hand side of the structural equation includes the exogenous variables and the endogenous variables estimated by the reduced-form equations. The model's development may be traced back to the pioneering work of Heckman (1978), and it is a common approach to deal with the endogeneity of binary-dependent variables. See Di Novi et al., (2020) and Di Novi et al. (2023) for applications that use the multivariate probit model to estimate a recursive system similar to the one used here.

8 We attempted an alternative specification in which the dummy variable indicating the presence of chronic conditions was replaced with a variable representing the number of chronic conditions. However, in this specific case, the model exhibited poorer performance: the continuous variable for chronic conditions was no longer statistically significant in the e-HISB equation, while remain significant in the other structural equations. This result, however, is in accordance to existing literature: indeed, what drives consumers to seek health information online is not so much the number of chronic diseases they suffer from, but rather whether they suffer from at least one chronic disease or not (see among the others Lee et al., (2014). or Bundorf et al., (2006).

9 One of the limitations of the second Corona survey database is that it does not allow for distinctions to be made between the type of health- related issues that respondents looked for on the Internet. To that end, we were unable to identify those who looked for general health information, health information related to COVID-19 pandemic, and information strictly related to a specific disease.

10 Healthcare systems experienced major disruptions during the initial months of the COVID-19 pandemic; this included restrictions, implied temporary closures of medical practices, and cancellation or postponement of non-emergency and elective procedures. The spread of information about the threat of the coronavirus exacerbated the fear of infection, especially among the elderly and chronically ill individuals, who, in several cases, forewent medical treatments (Di Novi & Santos, 2023).

11 This cycle of searches and anxieties can, in extreme cases, lead to a phenomenon known as cyberchondria which refers to the excessive or repetitive search for health information online, leading to increased levels of health anxiety or distress and an excessive use of healthcare services (Starcevic, et al., 2021).
